# Is political ideology correlated with brain structure? A preregistered replication

**DOI:** 10.1016/j.isci.2024.110532

**Published:** 2024-09-19

**Authors:** Diamantis Petropoulos Petalas, Gijs Schumacher, Steven H. Scholte

**Affiliations:** 1 Department of Psychology, The American College of Greece, Greece, Athens; 2 Department of Political Science, University of Amsterdam, Amsterdam, the Netherlands; 3 Department of Psychology, University of Amsterdam, Amsterdam, the Netherlands

**Keywords:** neuroscience, cognitive neuroscience, political science

## Abstract

We revisit the hypotheses that conservatism positively correlates with amygdala and negatively with anterior cingulate cortex (ACC) gray matter volume.Using diverse measures of ideology and a large and representative sample (Amsterdam Open MRI Collection [n = 928]), we replicate a small positive relationship between amygdala volume and conservatism. However, we fail to find consistent evidence in support of the ideology-ACC volume link. Using a split-sample strategy,we conducted exploratory whole-brain analyses on half the data, preregistered the findings, and then conducted subsequent confirmatory tests that additionally highlight weak, positive associations between the right fusiform gyri and conservatism. This is the largest preregistered replication study in the context of political neuroscience. By using Dutch as opposed to British or American data, we also extend the amygdala-conservatism link to a multiparty, multidimensional political context. We discuss the implications for future investigations of the neural substrates of ideology.

## Highlights

• We revisit a hypothesis linking ipolitical deology with gray matter volume in the amygdala and the ACC
• Using a large, representative sample only the ideology-amygdala hypothesis replicates
• Effect sizes of ideology on brain structure are very small
• Confirmatory tests link the left and right fusiform gyri with ideology

## Graphical abstract

**Figure:**
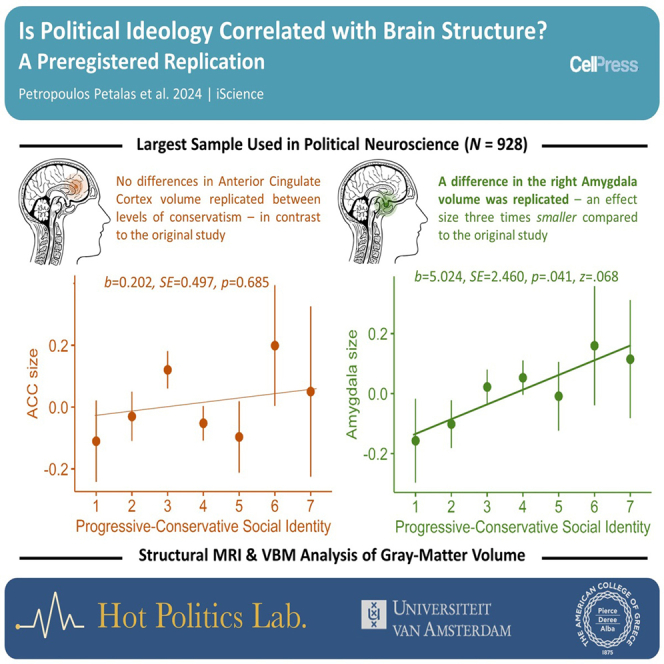

## Introduction

Do liberals and conservatives behave and think fundamentally different? This debate is ongoing with disagreement regarding ideological differences in negativity biases,^1^^,^^2^^,^^3^^,^^4^ cognitive rigidity, risk taking, and impulsivity.^1^^,^^5^ Underlying these differences is a fundamental claim first published in *Current Biology* that liberals and conservatives have identifiable differences in brain morphology. Specifically, Kanai et al.^6^ were the first to demonstrate that compared to liberals, conservatives show a larger volume of gray matter in the right amygdala (rAMG), a region that plays a critical role in the processing of negative, aversive human emotions like sadness, fear, and threat,^7^^,^^8^ and less gray matter in the anterior cingulate cortex (ACC), a region linked to error monitoring, belief updating, and affect-regulation.^9^^,^^10^^,^^11^

The study by Kanai et al.^6^ used magnetic resonance imaging (MRI) to investigate anatomical differences as a function of conservatism. The study sample consisted of (n = 90) UK university college students with a middle- to upper-class background and was accompanied by a small replication study using another (n = 28) participants from the same pool. Participants indicated their political orientation from 1 (very liberal) to 5 (very conservative) based on a single item.^12^ However, as there were no “very” conservative participants in the sample, the analyses were limited to the range of 1–4. Socioeconomic and demographic information and distribution of political orientation scores were not reported in the paper. Notably, the study by Kanai et al.^6^ was the first documented report focusing on morphological (i.e., structural) differences in gray matter volume between liberals and conservatives. As such, its originality has drawn scholarly attention. Hannah Nam et al.^13^ conducted a conceptual replication of the Kanai et al.^6^ hypotheses (i.e., correlation between gray matter volume and political ideology) in two independent samples of Caucasian (study 1, (n = 49) and ethnically diverse (study 2, n = 42) US college students, adding the general system justification scale (eight items) as a measure of perceived fairness, legitimacy, and justifiability of the prevailing social system (see the study by Kay and Jost^14^). Interestingly, although system justification was positively correlated with gray matter volume in the bilateral amygdala (and more so in the more diverse sample), self-reported ideology (single item) was not a significant predictor of amygdalar volume.

### Challenges in identifying neural correlates of political ideology

Taken together, these findings support an active role of the rAMG and the frontal lobes (in particular, the ACC) in processing threat and fear, social hierarchies, and social dominance, which is presumably the reason it correlates with conservatism. In both the Kanai et al.^6^ and the Nam et al.^13^ studies, however, ideology was simplified as a single-dimensional spectrum on conservatism, overlooking the multidimensional nature of political ideology. In addition, in both studies, the relatively small and rather homogeneous samples used limit the generalizability of the findings to the broader population.

Recent advances in neuroimaging further suggest that to reveal associations between the structure or function of the brain and observable characteristics or traits, known as brain-phenotype associations, large-scale neuroimaging data are required and that small-scale studies may be subject to type I error, due to the negative nature of the power law relation between required sample size and estimated effect size.^15^

Regarding the role of the amygdala in fear learning, existing evidence is inconclusive, with functional MRI (fMRI) studies yielding conflicting findings.^16^ This is problematic because the amygdala-fear relationship is typically seen as the primary theoretical link to conservatism.

Given that small samples undermine the probability of true positive results and that the evidence is mixed, replication studies using larger and more diverse samples across different sociopolitical landscapes are important. Such replications provide researchers with more accurate estimates of the relationships between brain structure and phenotype at the level of behavior and cognition. In addition, they allow researchers to evaluate if similar findings hold true across different populations. As such, the extent to which the morphological or structural properties of the amygdala drive political conservatism in more diverse and politically representative samples remains an important area of research in political psychology.

### Conceptualizing political ideology

There are three key conceptual issues with ideology. First, ideology can be seen as issue positioning, that is a coherent set of policy attitudes towards social and economic issues (e.g., “Homosexuals should be removed from society,” “Abortion should be illegal,” or “In general, you find society to be fair”) or as an identity—a political group or label to which one subscribes (e.g., Republican vs. Democrat in the US context). Although typically viewed as synonymous, ideological identification often does not align with ideology as policy attitudes.^17^^,^^18^^,^^19^ Second, ideology is often described as a single dimension running from liberal to conservative, or left to right. In ideologically homogeneous samples, the likelihood that single-dimensional measures can capture a general political orientation score is fair. In most democracies, however, one needs multiple dimensions to describe the variation in policy positions taken by parties and voters.^20^ Typically, researchers distinguish separate economic and social dimensions. How these two dimensions relate varies across time and between countries.^21^ Third, although there is evidence that party identification, self-reported ideology, and selected issue positions can vary over time within the same person, in the long run political attitudes appear to be rather stable.^22^

**Figure 1:**
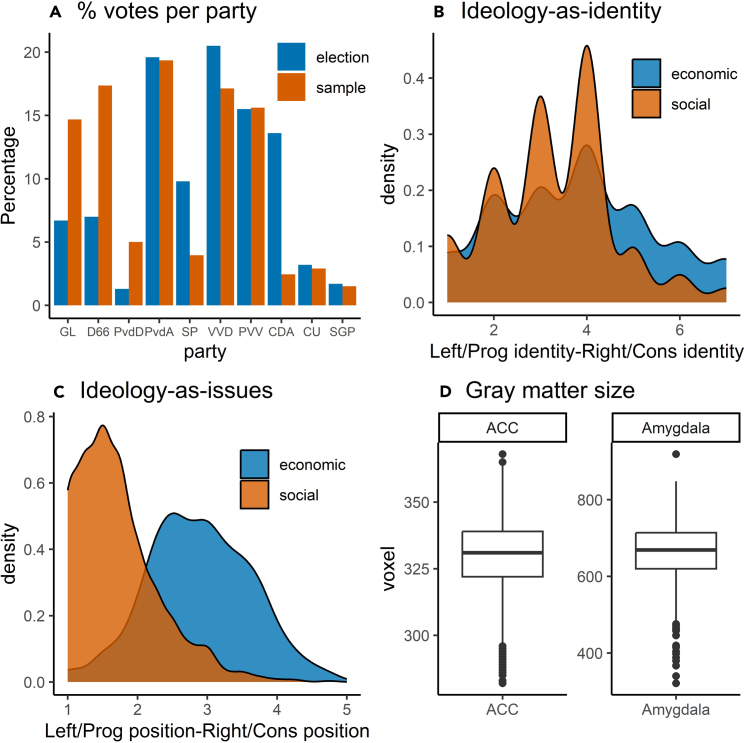
Sample distributions for ideology, vote choice, and gray matter volume in ACC and amygdala (A) Presents the percentage of voters for each party in the election and in the sample. (B) Shows the distribution of progressive-to-conservative identification (self-placement) on economic and social dimensions. (C) Shows the distribution of left/progressive-to-right/conservative issue positions on both the economic and social dimensions. (D) Shows boxplots to represent the distribution of gray matter volume in the ACC and amygdala.

To capture the complex relationships between political orientations and brain structure, we argue there is a need to distinguish between at least four different ideology constructs: social ideology as self-identification, economic ideology as self-identification, social issue positioning, and economic issue positioning (see Figure 1 for a visualization of our sample distributions for ideology and issue positioning). Comprehensive measures that encompass various ideology dimensions capture the complexity of political orientations, thereby providing a more nuanced understanding of the relationship between brain structure and specific political issues. For example, using more fine-grained measures of political orientation helps to identify the specific role of different neural regions in processing its different facets, such as party identification and issue alignment.

### The current study

Our study addresses these challenges by revisiting the original^6^ findings, using a larger, more diverse, and representative Dutch sample, using extensive measurement variation on ideology and vote choice, and taking into account the multifaceted and multidimensional nature of political ideology. By using Dutch as opposed to British or American data, we have data from a multiparty, multidimensional political context. This is in contrast to most studies published in the field, which are primarily based on a two-party political landscape. Thus, our study is designed to revisit and extend the long-assumed relationship between brain structure and political ideology.

We use structural MRI scans from the Amsterdam Open MRI Collection (see the study by Snoek et al.^23^), involving a total of (n = 928) healthy volunteers (mean age: 22.86 [SD = 1.70] years, 52% females), who were recruited between 2010 and 2012. We first replicate the same hypotheses as Kanai et al.^6^ They conceptualized ideology as ideology-as-identity. They hypothesized that the more conservative a person identifies, the more gray matter volume of the rAMG (H1); and the more liberal a person, the more gray matter volume of the ACC (H2). Then, we split the sample in half to conduct a whole-brain analysis and identified additional correlations between gray matter volume of various areas and our ideological constructs. We then preregistered the split half results, in addition to a few theoretically expected relationships, and conducted confirmatory tests on the second half of the data.

## Results

### Replication: Correlation amygdala and ACC volume with ideology

**Figure 2:**
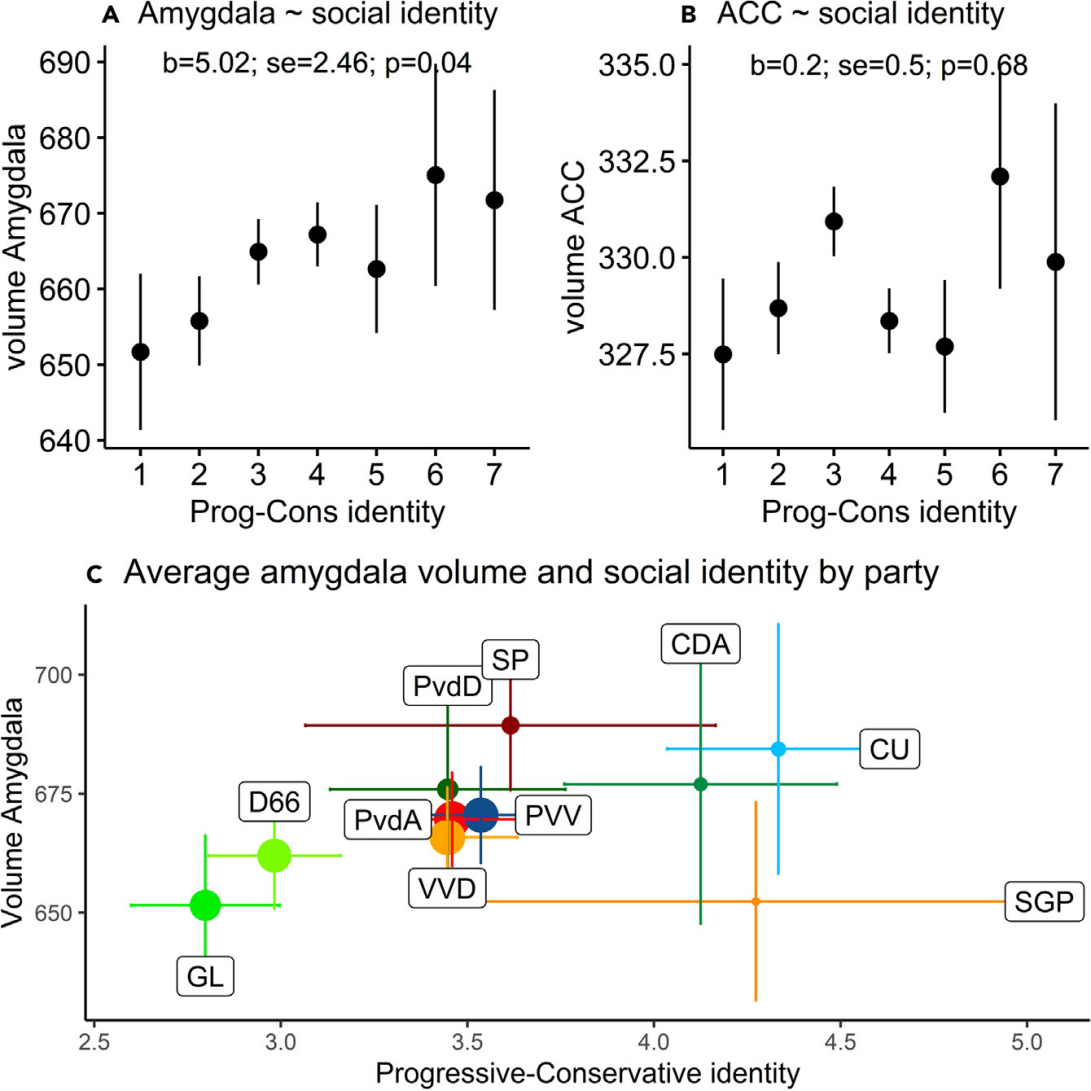
Relationships between ideological identitification, ACC, and amygdala (A and B) Show the means (dots) and 95% confidence intervals (bars) of respectively amygdala volume and ACC volume along the progressive-conservative social identity measure. The betas and SEs in (A and B) refer to a linear regression model with brain region as dependent variable and identity as a linear predictor. (C) Shows average amygdala volume and social identity per political party. The size of the dot of each party corresponds to the number of participants in our sample. The horizontal lines represent the 95% confidence intervals for identity, the vertical lines represent the 95% confidence intervals for amygdala volume.

We first replicate the two main findings from Kanai et al.^6^ Using OLS regression, we find a significant, positive effect of the social identity scale on amygdala volume (*b* = 5.024, *SE* = 2.460, *p* = 0.041, *z* = 0.068). Figure 2A plots this relationship. We fail to find a significant effect of the social identity scale on ACC volume (*b* = 0.202, *SE* = 0.497, *p* = 0.685, also see Figure 2B). In sum, we partially replicate the Kanai et al.^6^ findings. It should be noted, however, that our effect size is only a third of the one originally reported in the study by Kanai et al.^6^ (Pearson’s *R* of 0.068 compared to 0.23).

Figure 2C displays the means in social identity and amygdala volume per party. This figure confirms that voters of progressive parties such as D66 and GroenLinks (GL) have on average less gray matter volume in the amygdala region, and voters of more conservative parties have more gray matter volume in this region. SGP voters do not fit this pattern, which is most likely due to the small sample of SGP voters (*n* = 18).

Figure 2B also confirms the importance of taking into account multidimensionality; the economically radical-left socialist party has socially conservative voters and, on average, more gray matter in the amygdala. This sets them apart from other parties like GL and PvdA that are typically seen as “left-wing.” The Christian Union is economically centrist, but has deeply social conservative voters, also fitting the pattern of more gray matter in the amygdala. Again, this distinguishes this party from other parties that are identified as center-right on a unidimensional scale. Alternative operationalizations of social ideology or analyses of economic identity or ideology fail to identify a relationship with the amygdala (see Table S3) or ACC volume (see Table S4).

### Extension: Correlations between other areas and ideology

We subsequently conducted a whole-brain analysis on the first split-half of the data. In these analyses, we looked for a linear relationship between the volume of an area and each of the four ideology constructs adjusted for multiple comparisons. On this basis, we identified and preregistered a positive correlation between social ideology and the left (h3a) and right (h3b) fusiform gyrus (FG). Note that we did not preregister a link between social identity and the left FG, because the significant finding we had there was not robust against alternative model specifications. We further found and preregistered a negative correlation between social ideology and the ACC (h1). Note that there were minor voxel differences between our localization of the ACC and the one used in the study by Kanai et al.^6^ These preregistered expectations and an extensive pre-analysis plan were stored on OSF - https://osf.io/k7nt5/, prior to analyzing data from the second split-half.

**Figure 3:**
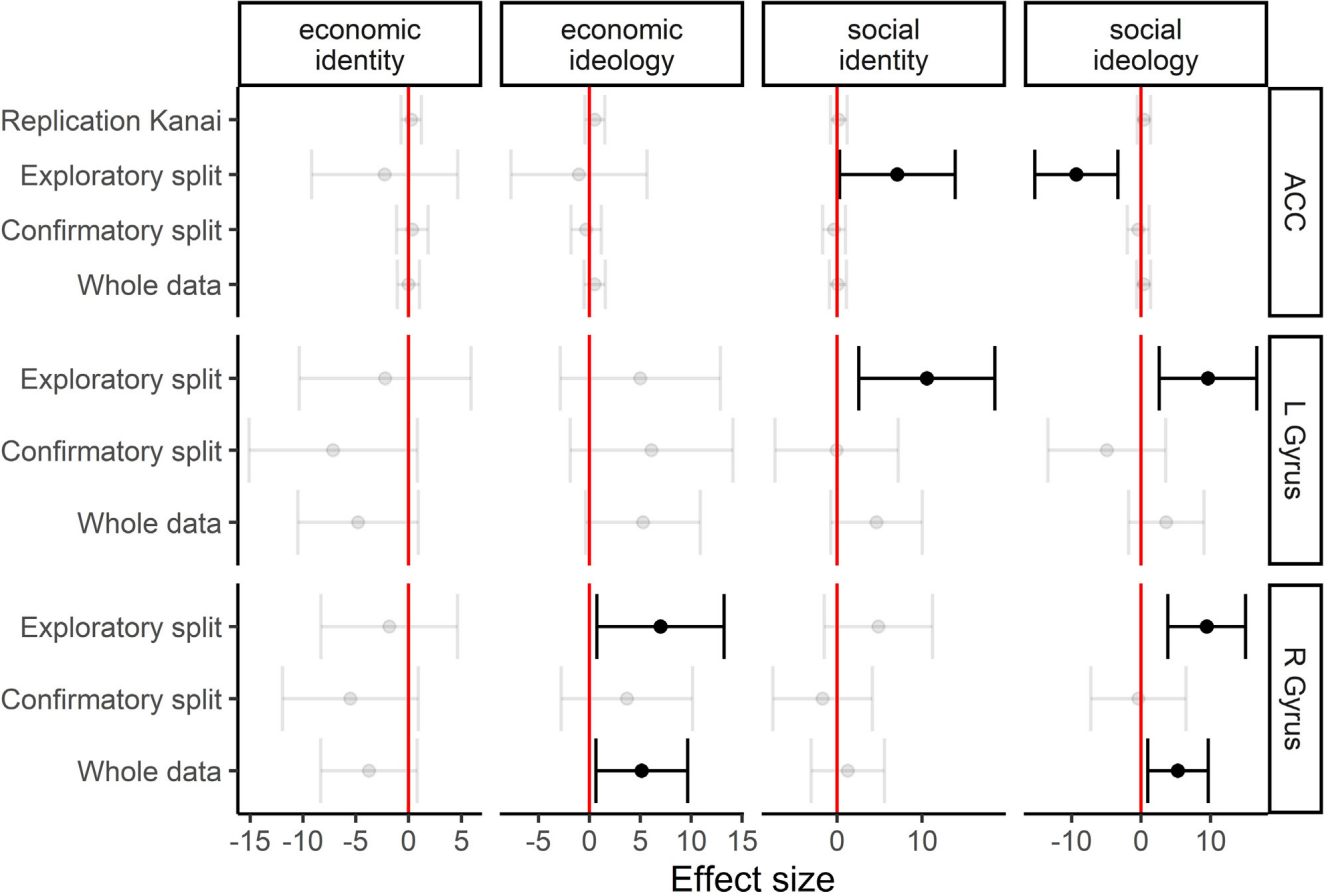
Ordinary least squares (OLS) regression coefficients of ideology on gray matter volume Each row represents a single analysis with the gray matter volume of a region as dependent variable, and the four ideology variables as independent variables. There is one exception: the original Kanai replication is based on four OLS regressions with single estimators. Dots represent the regression effect and bars the 90% CI. Black dots and bars indicate a significant relationship, gray ones denote an insignificant relationship.

Figure 3 presents the overview of these results. First, regarding the ACC (top panel), our exploratory analyses supported a negative relationship between social ideology and the ACC (Figure 3 also shows a positive effect of social identity. But this is driven by the modeling strategy. This effect disappears in a bivariate regression model, yet this was not replicated in our confirmatory part, nor in our analysis of the whole dataset. The Bayes factor of social ideology in the whole dataset analysis is 0.104, providing substantial evidence in favor of the null hypothesis of no effect. We therefore reject the link between social ideology and ACC.

Based on the exploratory analyses, we preregistered positive relationships between the volume of the left and right FG and social ideology. Both tests on the confirmatory split-half, however, find insignificant results as visualized in Figure 2. The whole data analysis indicates a positive, but insignificant relationship between social ideology and the left FG (whole data: *b* = 3.628, *SE* = 2.776, *p*, *one-tailed test* = 0.096, *z* = 0.044), as well as a positive and significant relationship between social ideology and the right FG (whole data: *b* = 5.298, *SE* = 2.221, *p*, *one-tailed test* = 0.009, *z* = 0.081).

To further assess the strength of our evidence, we calculated Bayes factors on the results of the whole data analysis. The Bayes factor of the social ideology-left FG link is 0.182, suggesting clear evidence in favor of the null hypothesis. We therefore reject hypothesis 3a. The Bayes factor for the social ideology-right FG link is still only 0.973, suggesting as much evidence for the null as for the alternative hypothesis. Placing more weight on our preregistered frequentist analyses than these post hoc Bayesian analyses, we conclude there is weak and suggestive evidence for a positive relationship between social ideology and the right FG. Yet, ultimately we need more data here.

Regarding economic ideology in the exploratory split, we find a statistically significant, positive relationship with the right FG. But we did not preregister this. The confirmatory split also finds a positive, but not significant, effect. The whole data analysis suggests a positive significant relationship (*b* = 5.147, *SE* = 2.298, *p*, *two-tailed test* = 0.025, *z* = 0.079). Also, here the Bayes factor (0.487) is substantially less favorable toward the alternative hypothesis than our preregistered frequentist analysis. Regarding economic identity, none of the results suggest a significant effect.

### Alternative explanations

To evaluate the robustness of our results and rule out alternative explanations, we ran a series of exploratory analyses. Specifically, we used social-economic background variables such as income, education, and gender; big-5 personality factors such as openness, conscientiousness, agreeableness, neuroticism, and extraversion; and the F-scale and the political-religious dimensions extracted from the Survey of Dictionary-based “Isms”^24^ as predictors of the volume of the key areas, namely the ACC, amygdala, left FG, and right FG. We selected these independent variables because they were included in the survey. We add these independent variables one-by-one to the analyses reported in Figure 3. Figure S1 shows the effect of the four ideology variables in models including the different independent variables we mentioned here. With one exception, none of the variables changes our main result, that is, each estimate of the effect of ideology is within the confidence interval of the main result. Only adding education changes the main result, but this is because we only have 349 observations for that variable, thereby dramatically reducing the sample.

## Discussion

The question whether biological characteristics play a role in political cognition goes back at least 50 years ago.^25^^,^^26^^,^^27^ The famous work by E.O. Wilson^28^ sparked initial interest among political psychologists and biologists, giving rise to the quest for specific genes^29^^,^^30^ and brain areas^31^^,^^32^ responsible for shaping political ideology. This has led to the emerging field of neuropolitics, more recently.^33^ Existing evidence partially supports a neurobiological association of the rAMG and ACC with political orientations, such that gray matter volume of the rAMG is greater in conservatives compared to liberals and that the ACC was larger in liberals.^6^^,^^13^^,^^34^

Adding to previous investigations, our study highlights that political orientation is a multi-dimensional concept. Large-scale neuroimaging data offer the possibility to test the robustness of previously reported brain-phenotype associations and to draw the big picture of individual differences and variation in measuring ideology. This has been particularly an issue in previously conducted investigations on this topic, which suffered from limited measures of ideology and used small and rather homogeneous sample sizes. Although still rare, such large-scale population MRI projects are important for advancing the field of neuropolitics.^35^^,^^36^ Here, we concretized ideology by dividing into an issue-based ideology measure and an identity-based measure, following political science work.^17^^,^^18^^,^^19^ We found that these measures are weakly and positively correlated, yet they also produce variation in how they relate to certain brain areas.

We first revisited the original Kanai et al.^6^ hypotheses and attempted a conceptual replication using a more diverse, large, and representative sample. Using a split-data design,^37^ we preregistered and replicated the finding that amygdala gray matter volume correlatives positively with conservatism. However, we failed to replicate the negative correlation between conservatism and the ACC. In all, given that the exploratory and the confirmatory data splits did not yield similar results, we should consider the analysis on the whole data as most informative. In addition, we analyzed gray matter volumetric associations with ideology and report correlations between economic and social dimensions of ideology and identity, and gray matter volume in the left and right fusiform gyri. Regarding the left FG, we fail to reject the null hypothesis of no relationship with any of the ideology concepts. However, we do find evidence that gray matter volume of the right part of the FG structure positively correlates with the economic and social dimensions of ideology (i.e., against governmental regulation and diversity/equality).

Notably, the effect sizes identified in our study are very small, so small that our exploratory and confirmatory findings do not align. In retrospect, this was to be expected because, with 450 participants, the power of a test that yields a Pearson’s *r* of about 0.08 is barely 52.1%. A correlation coefficient the size of 0.12 can only be detected at the acceptable power level of 80%. From that perspective, the analyses of all data pulled together deserve the most confidence. Even so, and considering the original study’s sample size, it is striking how much data were needed to replicate the Kanai et al.^6^ study (partially). This raises questions both about the replicability of much MRI research as well as the amount of underpowered null findings that are gathering dust in some drawer. It also suggests that reported effects heavily depend on different operationalizations of ideology, therefore yielding very low predictive power for political ideology on the basis of brain structure. In particular, social ideology, which was measured with issue-position items, is markedly skewed to the right, presumably because some of the items were too extreme for the Dutch context and because other social ideology as issue-position items, such as immigration or climate change, were not included in our questionnaire. For a comprehensive description of the social-ideology measure, see Table S1 in the STAR Methods section. This could explain the absence of a systematic effect for social ideology, suggesting that identification and issue alignment may be more fundamentally different than hitherto assumed and that it therefore matters which one of the two concepts researchers choose to analyze. Our findings suggest that a more nuanced operationalization of ideology is needed, compared to the oversimplified notion that liberals and conservatives have fundamentally different brains. While we do observe neurobiological differences in gray matter, it is critical to approach these findings with caution, to avoid fostering misconceptions and stereotypes, which can inadvertently fuel social polarization and undermine democratic participation and egalitarian values.

### Ideology and the amygdala

The amygdala is an almond-like structure located deep in the cerebral hemispheres of all vertebrates. In human adults, the amygdala contains approximately 13 million neurons. Based on its cytoarchitecture, the amygdala consists of six distinctive subregions, some of which have been previously linked to the regulation of mood and internal states. For instance, the basolateral complex and the centromedial group contain crucial subnuclei for processing pain sensitivity and emotional and social experiences, while dysfunctions to certain subregions have been linked to several psychiatric disorders (see the study by Zhang et al.^38^). The amygdala is a highly complex organ comprising a cluster of (at least) 13 nuclei in the rostral temporal lobe that are primarily involved in the processing of negative stimuli (like threats) and in regulating associated emotions, such as sadness, uncertainty, rage, and fear, this way efficiently serving cognitive and behavioral adaptations to environmentally salient events.^7^^,^^8^^,^^13^^,^^39^^,^^40^

Although the amygdala is part of the limbic system, an evolutionary old brain region, its structure and function are subject to developmental changes from adolescence to adulthood,^41^ as well as to situational factors such as chronic stress exposure.^42^ Research shows that mature neurons in the amygdala increase from childhood into adulthood.^39^ The distinctive characteristics of stress-induced changes in the amygdala, along with alterations in other brain regions, may have enduring effects on cognitive performance and the development of long-term anxiety.^38^ This could explain individual variation in amygdalar volume as a function of conservatism, as a regulatory response to perceived social treats.

In addition, there is evidence for a positive association between amygdalar gray matter volume and social status in macaques,^43^ socioeconomic status and learning of social hierarchies in humans,^44^^,^^45^ as well as social network size in both macaques and humans.^46^^,^^47^^,^^48^ This evidence aligns with human data showing that the amygdala is involved in the acceptance, learning, and maintenance of social hierarchies and social dominance,^13^ serving as another theoretical explanation for conservatism.

Combining findings from neuroimaging, brain lesions,^34^ and non-invasive brain stimulation studies enable direct observation of specific brain regions that are *necessary* for certain behavioral outcomes. Such studies may provide particularly convincing and methodologically rigorous insights regarding the relation between brain anatomy and political ideology or motivated reasoning. A recent study by Nam et al.^34^ compared political orientations in patients with frontal lobe and amygdala lesions and healthy control subjects. Results showed an association between conservatism and damage in the dorsolateral prefrontal cortex (DLPFC), but not the amygdala. While the role of the DLPFC in modulating conservatism has been further documented by Chawke and Kanai^49^ using repetitive transcranial magnetic stimulation, it is not possible to apply non-invasive stimulation methods to the amygdala, as it is a structure that lies deep in the brain.

In all, the implication of the amygdala specifically in predicting political orientations seems a consistent finding across multiple studies. The current replication study using a fairly large and politically diverse dataset adds partial support to this hypothesis. Our results imply that a progressive voter has on average an amygdala volume 0.14 standard deviation units smaller than a conservative voter does (we compare here the difference between one standard deviation below the social identity scale to one standard deviation above). This translates to 10 mm^3^ in physical space, which is just a little bit larger than a sesame seed. Of course, such a difference in the amygdala corresponds to thousands of neurons, possibly adding up to millions of neural synapses. We deem it possible that a small anatomical variation in the amygdala may link to substantial variance in functional orchestration and neural connectivity, accounting for some of the measurable differences at the level of political conservatism across individuals. However, we must not forget that the precise functions of the amygdala’s subnuclei and their role in fear learning remain elusive.^19^ Because of the correlational nature of neuroimaging studies, it remains unresolved if the association between amygdala volume and conservatism identified in the Kanai et al.^6^ study supports a causal role of the amygdala in regulating conservatism. In the pursuit of understanding the neural underpinnings of political ideology; we argue that future research in political neuroscience needs to shift toward functional connectivity of the amygdala and its subnuclei with other brain areas relevant for political conservatism.

### Politics and the FG

Located in the ventral temporal lobe’s center, the FG stands as the ventral temporal cortex’s most substantial component, distinguished by its intricate cytoarchitectonic properties and receptor distribution patterns. This region, essential for visual and cognitive functions, exhibits a sophisticated anatomical organization into medial (FGm), lateral (FGl), and anterior (FGa) subregions, each with unique connectivity signatures and roles.^50^ FGm primarily facilitates early visual processing, acting as a conduit to higher-order visual areas. In contrast, FGa specializes in processing semantic category information, such as faces or words, received from the FGl. Distinct functions emerge depending on the FG’s laterality, with the left FGl uniquely associated with language processing through fibers extending to related cortical brain regions, while visuospatial and face recognition tasks are processed bilaterally across the FG.^51^ Stimulation of right FGa selectively impairs the ability to recognize face identity.^52^ This anatomical and functional specificity underscores the FG’s role in decoding visual information along the ventral temporal lobe’s posterior-anterior axis, highlighting its critical involvement in categorical perception of faces, discrimination of body parts, recognition of object features, and semantic processing.

Research in political neuroscience has indicated a functional role of the FG in processing politically salient stimuli, including faces and statements, as well as non-political stimuli depending on preexisting political attitudes.^53^ In a recent electroencephalogram (EEG) study,^54^ resource-scarcity framing was found to reduce FG activity to Black (vs. White) faces, suggesting biases in visual processing of identity-related characteristics under conditions of economic stress. A 2020 fMRI study found that individuals with xenophobic (versus non-xenophobic) attitudes elicited higher BOLD responses to faces of refugees and terrorists in the left FG and increased functional connectivity between the distinct clusters of the FG and the right middle frontal gyrus, the left supplementary motor area, the right precuneus, and the right lingual, parahippocampal gyri.^55^ Furthermore, the FG is involved in partisan-dependent neural synchronization in response to political videos.^56^ These findings suggest that information processing in the FG reflects not only the detection of low-level visual features but also the dynamic computation of high-level aspects of the stimulus, including social categories, stereotypes, and attitudes, in a top-down manner.^57^

In our study, we find a positive relationship between gray matter volume in the right FG structure and issue alignment with statements relevant to economic and social attitudes, indicating a nuanced role of the right part of the FG in processing specific statements tied to social and economic concepts. In all, and given that the FG’s anatomical specialization and connectivity with other brain regions support complex semantic processing and biased encoding of political information, we deem that findings from our study reinforce the notion that structural differences in the FG may underpin higher-order cognitive processes related to political orientation. This suggests a sophisticated neural basis for political attitudes, rooted in how the FG integrates and interprets complex visual input bearing social significance. This prediction should be addressed further in future studies, using functional neuroimaging methods such as EEG or fMRI, in order to avoid interpretations based on reverse inference.

### The ACC and other areas

The ACC lies in the medial wall of each hemisphere, close to the corpus callosum. It is also a very complex organ linking to multiple areas of the brain, including direct connections to the limbic system and the amygdala. Based on its cytoarchitecture, receptor mapping, and connections, the ACC can be separated into a ventral- and a middle-cingulate cortex area. In brief, the middle cingulate cortex shows high connectivity with cognitive- (e.g., lateral prefrontal) and motor-related (e.g., premotor and primary motor) areas of the cortex and with both pain- and motor-related thalamic nuclei. The ventral part of the ACC has extensive connections with areas related to emotion (e.g., amygdala), memory (e.g., hippocampal region), and reward (e.g., orbitofrontal cortex and ventral striatum), with specialized neurons that enable faster signaling, thereby facilitating action execution and adaptation in social situations.^11^

The ACC is primarily involved in conflict detection,^58^ and political conservatism can be seen as increased sensitivity to conflicting information relative to traditional values. In the original Kanai et al.^6^ study, the ACC was found to be larger in liberals. In our study, we find inconsistent evidence that gray matter volume in the ACC correlates with conservatism. Results from the exploratory and the preregistered confirmatory sections of our data do not align. Although our study was sufficiently powered, we still fail to replicate this relationship.

Nonetheless, and given that the ACC has been linked to the detection of conflicting ideological principles in previous investigations,^31^^,^^59^^,^^60^ it is possible that differences between liberals and conservative spotted in the ACC can reflect functional instead of structural properties. Functional neuroimaging and connectivity studies, focusing on functional rather than on anatomical properties, have also documented differences in the ACC and in other brain regions, between liberals and conservatives.^13^^,^^31^^,^^36^^,^^61^^,^^62^ Recently, Yang et al.^36^ presented a functional connectivity analysis of 174 liberal and conservative brains, using self-reports and behavioral paradigms used in cognitive neuroscience to tackle human performance in fundamental cognitive domains (i.e., retrieval, empathy, and monetary reward). Using convolutional neural networks analysis, the researchers modeled a functional connectivity network that predicts political conservatism, linking the rAMG, inferior frontal gyrus, and hippocampus together.

In addition to these regions, differences between liberals and conservatives have been previously linked to patterns of neural activation in the left insula,^27^^,^^32^^,^^63^ the ventromedial, dorsolateral, and dorsomedial parts of the prefrontal cortex,^34^^,^^49^^,^^64^^,^^65^^,^^66^^,^^67^ the dorsal part of the ACC,^68^ the temporoparietal junction, and the precuneus.^69^ Activity in these brain regions typically corresponds to functions like motivation and disgust,^70^^,^^71^ emotional processing, decision-making, memory, self-perception, and social cognition,^72^ attention and inhibition, reward processing, mentalizing (i.e., thinking about the mental states of the self and others), error monitoring, and processing of pain. Because all these regions are involved in a diverse array of cognitive functions, associations with ideology should be regarded with caution and call for further investigation. In our whole-brain analysis, we did not find relationships between these areas and the different ideology concepts we tested for.

### Final remarks: Brain morphology and political orientations

The ability to determine the specific regions of the brain that engage during political judgment and decision-making creates a foundation for examining the differing worldviews of liberals and conservatives. This perspective assumes that the complex components of partisanship and ideology can either (1) be deducted to morphological differences in a small set of brain regions, implying that an independent module or network with its own domain-specific functions and properties determines political orientations or (2) be attributed to functional connectivity networks, implying that ideological beliefs are emergent mental constructs that arise from the dynamic interplay between low-level feature processing and higher-order cognitive operations relevant for social categorization, identification, and valuation.^5^^,^^55^^,^^73^ The integration of specific question items related to social identity, economic identity, social ideology, and economic ideology into demographic surveys for large neuroimaging projects presents an opportunity to advance political neuroscience. Such granularity is essential for a comprehensive understanding of the complex relationship between brain structure and political beliefs, providing valuable insights into the cognitive underpinnings of ideology.

## STAR★Methods

### Key resources table

**Table:** 

REAGENT or RESOURCE	SOURCE	IDENTIFIER
**Software and algorithms**		

R	CRAN	https://cran.r-project.org/
FSL	FreeSurfer v6.0.1	Dale et al., 1999^28^
ANTs v2.1.0	antsRegistration tool	
4BiasFieldCorrection v2.1		
antsBrainExtraction.sh v2.1		

**Other**		

Philips 3T	Philips	https://www.usa.philips.com/healthcare/resources/landing/the-next-mr-wave/ingenia-elition

### Resource availability

#### Lead contact

Further information and requests for resources should be directed to and will be fulfilled by the lead contact, dr. Diamantis Petropoulos Petalas (dpetropoulos@acg.edu).

#### Materials availability

This study did not generate new materials.

#### Data and code availability

• All original MRI data can be accessed from the Amsterdam Open MRI Collection (see Snoek et al.^23^).
• Data reported in this study can be accessed in a public repository containing the original (raw) and preprocessed data: https://openneuro.org/datasets/ds003097/versions/1.2.1 lead contact
• The code used to analyses the data an be accessed through Open Science Framework: https://osf.io/k7nt5/
• Any additional information required to reanalyze the data reported in this paper is available from the lead contact upon request.

### Experimental model and participant details

We use structural magnetic resonance imaging scans from the Amsterdam Open MRI Collection (see Snoek et al.^23^), involving a total of (*N* = 928 healthy volunteers (mean age: 22.86 (SD = 1.70) years, 52% females), who were recruited via an agency (Motivaction international B.V.) between 2010 and 2012. The STAR Methods section A provides more details regarding the scanner and protocol.

#### Sample description

The sample is representative of the Dutch population in terms of educational level (low: 10%, medium: 43%, high: 43%) and socioeconomic status (very low: 16%, moderately low: 26%, average: 28%, moderately high: 19%, high: 11%). Figure 1 shows that the voting preferences of our sample are more left-wing or liberal than the general population. A rough distinction between left-wing and right-wing parties yields 40% of sample participants favoring the right, whereas this is 55% in the population. Yet radical parties such as the SGP, PVV, and Christian Union are very well represented in the sample, indicating sufficient variation in ideology.

#### Procedure

Written consent was obtained from all participants and the study was approved by the faculty’s ethical committee prior to data collection (EC number: 2010-BC-1345). All participants were informed upfront about the goal and scope of the research, the MRI protocol, safety measures, general experimental procedures, privacy and data sharing concerns, and voluntary nature of the project (i.e., participants were explained they could withdraw from the study at any time, without giving a reason for it).

### Method details

#### Measuring ideology

To measure ideological identification on the social dimension, we used a single-item that asked participants to indicate on a 1–7 Likert scale if they identify themselves politically progressive/conservative (social identity). To measure ideological identification on the economic dimension we used a similar question that asked participants to identify themselves as economically left/right-wing (economic identity). On average, respondents scored moderately in the two orientation constructs (raw score left/right-wing: *M* = 3.79, *Mdn* = 4, *SD* = 1.64; raw score progressive/conservative: *M* = 3.31, *Mdn* = 3, *SD* = 1.3) as is also shown in Figure 1B. In the analyses these two variables are z-standardized to facilitate interpretation.

We measured economic ideology based on the following four items, using a Likert scale from 1 to 5: ’Are you for or against the government taking drastic measures to reduce the differences in possession?’, ’Are you for or against the government taking drastic measures to reduce the differences in ownership?’, ’Are you for or against the government taking drastic measures to reduce the differences in income?’; and ’The government must/oblige companies to allow employees to share in the profits as much as they do’. With a Cronbach’s alpha of 0.73 economic ideology has an acceptable fit.

The grand mean of these items is 2.88 which indicates a minor skew to the left (also see Figure 1C We measured social ideology by combining scores from the following four items: ’Homosexuals should be removed from society’, ’Homosexuals should be freed as much as possible to live their own way’, ’It is unnatural for women to lead men in a company’; and ’Women are more suitable for raising small children than men’. Social ideology had a weak fit (Cronbach’s alpha of 0.6) due to the fact the data is highly skewed, as shown in Figure 1C. Ideally, social ideology would also include questions on immigration and climate change. Nonetheless, with SGP and Christenunie voters the most socially conservative on this scale and Groenlinks the most socially progressive, the scale does have face validity. Both the social ideology and economic ideology variables are z-standardized (Table S1).

#### Scanner details and protocol

All participants were scanned on the same Philips 3T (Philips, Best, the Netherlands), using a 32-channel head coil. At the beginning of each scan session, a low-resolution survey scan was made, which was then used to determine the field-of-view location. For all structural (T1-weighted) scans, the slice stack was not angulated. The set of scans acquired was relatively consistent across all three scan sessions and participants.

High-resolution anatomical images were acquired using a T1-weighted 3D Modified Driven Equilibrium Fourier Transform sequence (repetition time = 8.10 ms; echo time = 3.70 ms; field of view = 160∗256∗256 mm; voxel size = 1∗1∗1 mm; saggittal acquisition direction).

#### MRI and voxel-based morphometry (VBM) preprocessing steps

Each T1w MR image volume was corrected for intensity non-uniformity using 4BiasFieldCorrection v2.1.0^74^ and skull-stripped using antsBrainExtraction.sh v2.1.0 (using the OASIS template). Brain surfaces were reconstructed using recon-all from.

FreeSurfer v6.0.1,^75^ and the brainmask estimated previously was refined with a custom variation of the method to reconcile ANTs-derived and FreeSurfer-derived segmentations of the cortical gray-matter of Mindboggle.^76^ Spatial normalization to the ICBM 152 Nonlinear Asymmetrical template version 2009c^77^ was performed through nonlinear registration with the antsRegistration tool of ANTs v2.1.0,^78^ using brain-extracted versions of both T1w volume and template. Brain tissue segmentation of cerebrospinal fluid (CSF), white-matter (WM) and gray-matter (GM) was performed on the brain-extracted T1w using fast.^79^ These files were then registered to the MNI152 standard space using non-linear registration.^80^ The resulting images were averaged and flipped along the x axis to create a left-right symmetric, study-specific gray matter template. Second, all native gray matter images were non-linearly registered to this study-specific template and “modulated” to correct for local expansion (or contraction) due to the non-linear component of the spatial transformation.
