# Supplementary material for: Is political ideology correlated with brain structure? A preregistered replication
Source: iScience. 2024 Sep 19;27(10):110532. doi: 10.1016/j.isci.2024.110532 (PMC11519434; doi:10.1016/j.isci.2024.110532)
Supplement: Document S1. Figure S1 and Tables S1–S4 [file mmc1.pdf]

iScience, Volume *27*

Supplemental information

Is political ideology correlated with brain structure? A preregistered replication

Diamantis Petropoulos Petalas, Gijs Schumacher, and Steven H. Scholte

Table S1

Summary Statistics

Variable	N	Mean	Std. Dev.	Min	Pctl. 25	Median	Pctl. 75	Max
Homosexuals should be removed from society	964	1.2	0.64	1	1	1	1	5
Homosexuals should live their life as they please (R)	964	1.5	0.88	1	1	1	2	5
Unnatural for women to lead	964	1.5	0.89	1	1	1	2	5
Women should raise children	964	2.6	1.2	1	2	3	4	5
Differences in possession	964	3.1	0.96	1	2	3	4	5
Differences in income	964	2.9	1.1	1	2	3	4	5
Sharing of profit	964	3	0.99	1	2	3	4	5
Taxation of higher incomes	964	2.5	0.93	1	2	2	3	5

Table S2

Parties and vote choice in the sample and in the population

Party name	Description	Social ideology (0-10)	Economic ideology (0-10)	Vote choice 2010	Sample
CDA	Christian democratic	7.1	6.5	13.6%	2.2%
Christenunie	Christian social	7.4	5	3.2%	2.6%
D66	Social-liberal	1.6	5.5	7%	16.9%
Groenlinks	Green	1.9	3	6.7%	13.8%
PvdD	Animal-rights	3.8	3.6	1.3%	5.1%
PvdA	Social democratic	4	3.6	19.6%	18.4%
PVV	Radical right	7.2	5.2	15.5%	15.8%
SGP	Christian conservative	9.3	6.5	1.7%	1.5%
SP	Socialist	5.2	1.5	9.8%	4.1%
Trots	Radical right	na	na	0.6%	0.4%
VVD	Conservative-liberal	4.8	8.3	20.5%	17.1%

Note: the social and economic ideology and the vote choice variables stem from the Chapel Hill Expert Survey[11]; Sample refers to vote choice from participants in our study.

Table S3

OLS regression coefficients predicting amygdala gray matter volume

	(1)	(2)	(3)
Social identity	5.024*	5.135*	
	(2.460)	(2.474)	
Economic identity		−1.093	
		(2.457)	
Social ideology			−2.125
			(2.442)
Economic ideology			1.029
			(2.477)
Constant	663.250***	663.270***	663.288***
	(2.457)	(2.459)	(2.464)
Observations	900	900	900
R^2	0.005	0.005	0.001
Adjusted R^2	0.004	0.003	−0.001
Residual Std. Error	73.720 (df = 898)	73.753 (df = 897)	73.896 (df = 897)
F Statistic	4.170* (df = 1; 898)	2.182 (df = 2; 897)	0.450 (df = 2; 897)

Note: The first model is the basic replication. The second model is a robustness check including the economic ideology-as-identity variable. The third model includes both ideology-as-issues variables. We opted here a single model including both variables rather than two separate models, because the effect sizes are nearly similar, and always insignificant. $^{*}p<0.05$; $^{**}p<0.01$; $^{***}p<0.001$

Table S4

OLS regression coefficients predicting ACC gray matter volume

	(1)	(2)	(3)
Social identity	0.202	0.179	
	(0.497)	(0.500)	
Economic identity		0.227	
		(0.497)	
Social ideology			0.383
			(0.492)
Economic ideology			0.523
			(0.499)
Constant	329.130***	329.126***	329.121***
	(0.497)	(0.497)	(0.497)
Observations	900	900	900
R^2	0.0002	0.0004	0.002
Adjusted R^2	−0.001	−0.002	−0.0003
Residual Std. Error	14.902 (df = 898)	14.908 (df = 897)	14.897 (df = 897)
F Statistic	0.165 (df = 1; 898)	0.187 (df = 2; 897)	0.887 (df = 2; 897)

Note: The first model is the basic replication. The second model is a robustness check including the economic ideology-as-identity variable. The third model includes both ideology-as-issues variables. We opted here a single model including both variables rather than two separate models, because the effect sizes are nearly similar, and always insignificant. $^{*}p<0.05$; $^{**}p<0.01$; $^{***}p<0.001$

Figure S1

OLS regression coefficients of ideology on area volume

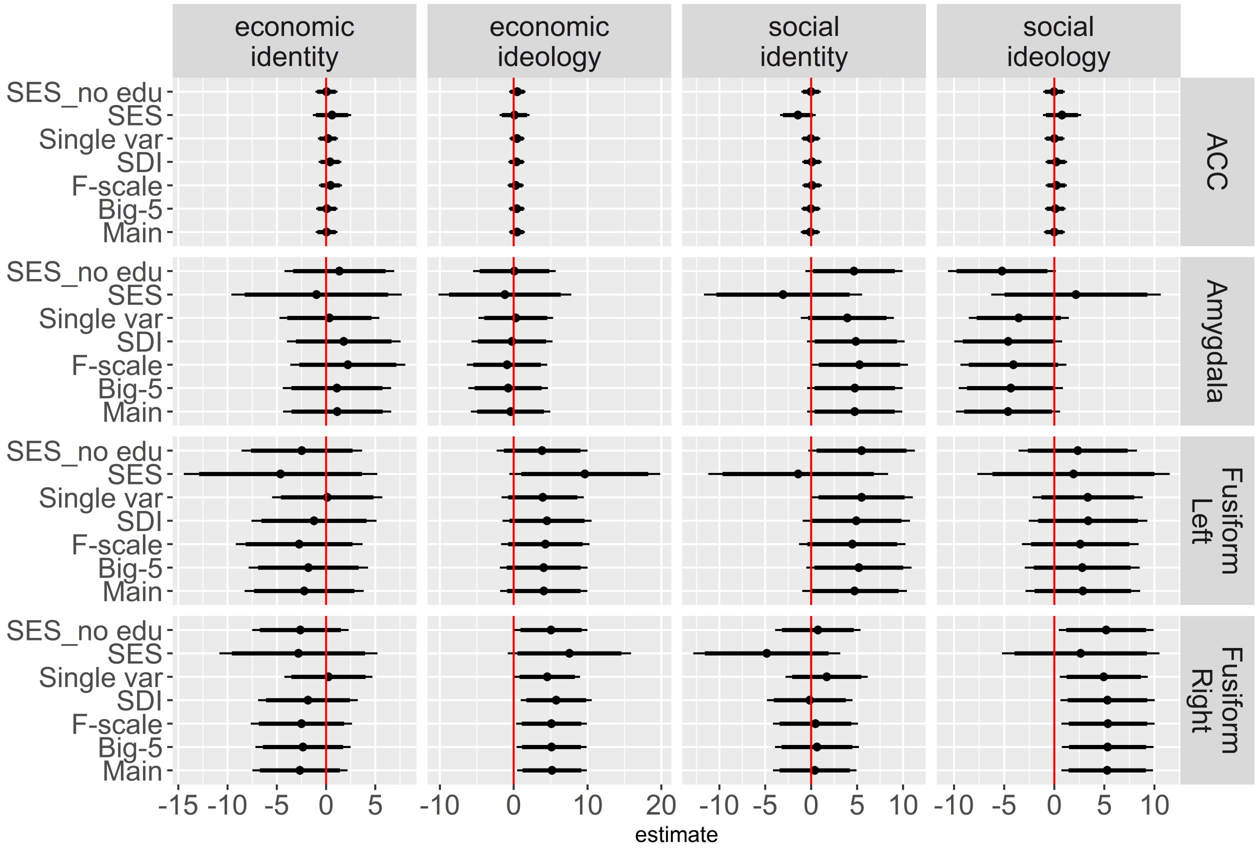

Note: Each row represents a single OLS regression. The row label indicates which variables we add to the basic equation of volume area = social identity + social ideology + economic identity + economic ideology. "SES" stands for socio-economic background variables (education, income and gender). With "SES no edu" we remove education because we only have 349 observations for that variable. The label "single var" is different, here we ran the analyses per area with only one independent variable. Main represents the main analysis reported in the paper.
